# Lactate-related metabolic reprogramming and immune regulation in colorectal cancer

**DOI:** 10.3389/fendo.2022.1089918

**Published:** 2023-01-26

**Authors:** Qianhui Sun, Jingyuan Wu, Guanghui Zhu, Tingting Li, Xiaoyu Zhu, Baoyi Ni, Bowen Xu, Xinyi Ma, Jie Li

**Affiliations:** ^1^Oncology Department, Guang’anmen Hospital, China Academy of Chinese Medical Sciences, Beijing, China; ^2^Graduate College, Beijing University of Traditional Chinese Medicine, Beijing, China

**Keywords:** lactate, colorectal cancer, immunoregulation, metabolic reprogramming, immune response

## Abstract

Changes in cellular metabolism involving fuel sources are well-known mechanisms of cancer cell differentiation in the context of carcinogenesis. Metabolic reprogramming is regulated by oncogenic signaling and transcriptional networks and has been identified as an essential component of malignant transformation. Hypoxic and acidified tumor microenvironment contributes mainly to the production of glycolytic products known as lactate. Mounting evidence suggests that lactate in the tumor microenvironment of colorectal cancer(CRC) contributes to cancer therapeutic resistance and metastasis. The contents related to the regulatory effects of lactate on metabolism, immune response, and intercellular communication in the tumor microenvironment of CRC are also constantly updated. Here we summarize the latest studies about the pleiotropic effects of lactate in CRC and the clinical value of targeting lactate metabolism as treatment. Different effects of lactate on various immune cell types, microenvironment characteristics, and pathophysiological processes have also emerged. Potential specific therapeutic targeting of CRC lactate metabolism is also discussed. With increased knowledge, effective druggable targets might be identified, with the aim of improving treatment outcomes by reducing chemoresistance.

## 1 Introduction

Colorectal cancer (CRC) is the second most common adult cancer in females and the third most common in males. It is the fourth leading cause of cancer-related death, accounting for 9.2% of deaths worldwide (1, 2), and is considered a global health concern. Despite consistent improvements in morbidity and mortality with the application of chemotherapy and targeted therapies, there is an urgent need to develop novel effective treatment strategies (3). Human tumor cells and their structures have important complex of metabolic patterns with marked heterogeneity (4, 5). The concept of “metabolic reprogramming” refers to the reprogramming metabolic pathways in the presence of mutations responsible for the initiation of cancer to allow developing tumor cells to acquire metabolic properties that support cell survival, escape immune surveillance, and hyperplastic growth. It is considered to be an essential component of malignant transformation, and its unqiue metabolic phenotype is associated with tumor genotype, tumor progression and metastasis (6–8). “Metabolic reprogramming” is regulated by oncogenic signaling and transcriptional networks to alter the metabolic patterns of glucose, amino acids, lipids, and nucleic acids through processes such as aerobic glycolysis, glutamine catabolism, macromolecular synthesis, and nuclear acid homeostasis (4, 9). In the context of carcinogenesis, alterations in cellular metabolism involving fuel sources (glucose, fatty acids, etc.) have emerged as an important mechanism for the differentiation of cancer cells (10). Abnormal metabolites or intermediates of tumor metabolism play an important role in regulating the proliferation, differentiation, activation and function of immune cells (11–13). Its metabolism is highly dynamic, responding to both intracellular and extracellular effects (7), and could affect the immune system, which is closely related and collectively referred to as “immune metabolism” (14).

The hypoxic tumor microenvironment induces the glycolytic phenotype of many cancer cells, and glycolysis and mitochondrial oxidative phosphorylation(OXPHOS) jointly maintain the balance of energy metabolism in cancer cells (15). Cancer cells still prefer to utilize glycolytic functions rather than OXPHOS, even under normoxic conditions. This phenomenon is the Warburg effect, also known as aerobic glycolysis, which is a crucial component of metabolic reprogramming in most tumors (16). A variety of malignant tumors, including CRC, have been found to undergo glycolysis at a higher rate than non-tumor tissues. The metabolic regulation and alteration of cancer cells are complex. The energy obtained from glycolysis is conducive to the proliferation, invasion, migration, and epithelial-mesenchymal transition(EMT) of cancer cells (17). The mechanisms leading to this “selfish” metabolic reprogramming may be related to the Hypoxia-inducible factor-1(HIF-1) overexpression (18), activation of oncogenes such as c-Myc and Ras, and PI3K/Akt/mTOR pathways (19–25). Oncogenic mutations and proto-oncogene expression can enhance the activity of certain metabolic enzymes to acquire and maintain sufficient nutrients to meet synthetic and metabolic requirements at the edge, leading to metabolic reprogramming of cancer cells. The high expression of c-Myc increases the level of metabolic enzymes and enhances glycolysis, glutaminolysis, nucleotide metabolism, and fatty acid synthesis (21–23). Mutated Ras and c-SRC may assist cancer cells in transporting extracellular proteins and cell debris to phagocyte more amino acids and lipids (20). K-Ras can directly regulate the conversion of glycolytic enzymes such as Hexokinase 1 (HK1) to cause mitochondrial dysfunction, enhance glycolysis, and promote the occurrence of tumors in colorectal and pancreatic tissues (26, 27). In addition, some differentially expressed lincRNAs could be regulated by specific molecular signaling pathways to promote cellular apoptosis or inhibit cellular proliferation in CRC cells *via* the Warburg effect (28, 29). One of the final metabolites in the aerobic glycolysis process of tumor tissue is lactate.

As a relatively common and ancient signaling molecule, lactate easily accumulates in the tissue microenvironment, leading to lactic acidosis (30). Additionally, lactate may regulate metabolic pathways, immune responses and intercellular communication in the tumor microenvironment, and has a wide range of physiological functions in carbon metabolism, cell signaling and epigenetic regulation (20, 29–31). Elevated levels of VEGF and arginase are involved in metabolism and immune regulation in the tumor microenvironment, with the acidification of lactic acid caused by excessive glycolysis. Lactate mediates the polarization of Mφ toward pro-tumor phenotypes. Classically activated Mφ regulates metabolic and inflammatory phenotypes by promoting the production of lactate in glycolysis mediated by the AKT/mTOR/HIF-1α pathway, and their alternate activation has different pathway changes (31–34).

In this review, we summarize the latest studies on lactate as a metabolite and signaling molecule of glycolysis, which promotes metastatic progression of CRC through metabolic reprogramming, activation of immune cell interactions, and effects on epigenetic modifications of immune cells from oncological and immunological perspectives. More importantly, their implications for future study are evaluated, and potential strategies targeting lactate metabolism are proposed to break through the restriction of current therapies.

## 2 Effect of lactic acid on immune cells in the tumor microenvironment of CRC

**Figure 1:**
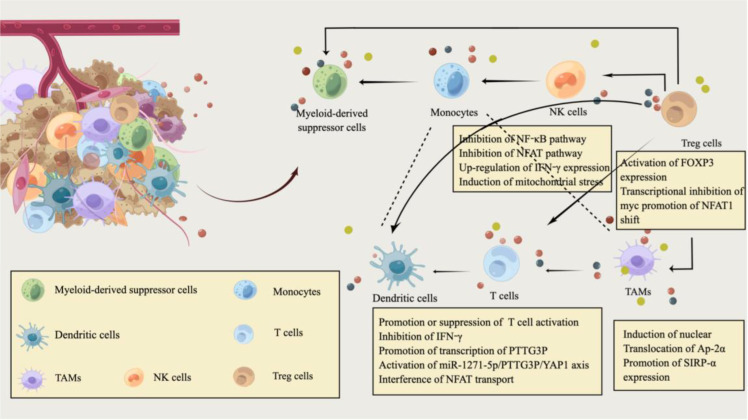
Immune modulation by lactate in the tumor microenvironment of CRC. The tumor microenvironment (TME) of CRC is filled with various cell populations. In addition to CRC cells, there are a variety of immune cells and tumor stromal cells. Similarly, the metabolic reprogramming of CRC cells is also inseparable from the regulation of the immune system, and immune metabolism plays an essential role in the pathogenesis and progression of CRC. CRC cells consume most nutrients and secrete excessive lactate into the CRC extracellular microenvironment. Hypoxic and lactate-acidified TME have specific immunosuppressive properties. Lactate also regulates the metabolism of innate and adaptive immune cells by affecting the function of T cells, natural killer (NK) cells, and dendritic cells(DC). Lactic acid can attract Treg cells and help Foxp3^+^ regulatory T (Treg) cells to maintain their immunosuppressive function in an acidic environment. In addition, lactate, as an extracellular molecule regulating tumor-associated macrophages(TAM) polarization, affects the phagocytic activity and carcinogenic effect of TAM by promoting Sirpα expression. This image was designed by Qianhui Sun and drawn by Figdraw. All authors confirm the originality of it and retain copyright to it.

It is well known that the immune system contains a variety of immune cells, such as monocytes, macrophages, dendritic cells(DCs), lymphocytes, and natural killer cells (NK cells) (35). The resting immune system can be activated to respond to external stimuli such as inflammation or infection (36), and immune cells in different states may have different metabolic patterns to meet changing demands for energy use (37, 38). In addition to being present in cancer cells, the shift in metabolic patterns can be a feature of the rapid proliferation of other immune cells (37). Immune cells in the TME may be accompanied by different degrees and types of cancer cell infiltration. Cancer cells can inhibit the antitumor effect of immune cells by competing and consuming nutrients necessary for metabolism or reducing the metabolic fitness of immune cells in the tumor microenvironment by other methods (39, 40). More importantly, glucose is an essential nutrient for immune cells to proliferate and function (41, 42). Like cancer cells, TILs require nutrients in the TME to support their proliferation and differentiation and further participate in the immune response (43). As confirmed in previous studies, lactic acid plays a vital role in the metabolic coupling of cancer-associated fibroblasts(CAFs) and cancer cells. It targets and inhibits CAFs glycolysis through different metabolic patterns to reduce the invasion and metastasis of tumor cells (44). Both lactate-mediated intracellular signaling and nuclear induction are all involved in this regulation (**Figure 1**).

### 2.1 T cells

T cells are involved in the immune regulation of almost all tumors. They play an essential role in the pathogenesis and progression of CRC (45), mainly including helper CD4^+^T cells and cytotoxic CD8^+^T cells. The differences in the activation status and types of T cells may be closely related to the prognosis of CRC patients (46–48). The activation of CD4^+^T cells by antigen-presenting cells promotes the migration of highly cytotoxic CD8^+^T cells to the tumor site and plays the role of tumor immune mediators (49). Exhausted CD8^+^T cells have abundant high expansion potential in TME (48). As a common metabolite, lactate can be rapidly produced by both tumor cells and activated T cells, but its function is not unchanged. Due to the heterogeneity of the microenvironment, it may have different effects on the activation of T cells and antitumor immunity. An example is that lactic acid can inhibit the activity of histone deacetylase, leading to the increase of H3K27 acetylation at the Tcf7 super-enhancer site and the increase of Transcription factor 7(Tcf7) gene expression to induce the stemness of CD8^+^T cells and enhance the anti-tumor immunity (50). Notably, Galon studied samples from about 400 patients and concluded that CD8 and CD45RO T cells at the tumor core were better prognostic factors in CRC than traditional clinical staging (49). Lactic acid(LA) promoted the expression of CXCL10 and Cadherin-11 in CD115^+^ precursor cells through the PI3K-AKT pathway, and CXCL10 stimulates the recruitment of CD4^+^ T cells to the metastatic site and induces RANKL production, leading to CRC bone metastasis (51, 52).

On the other hand, lactic acidosis can not only promote the activation of T cells but also inhibit the activation of P38 and JNK/c-JUN pathways triggered by T cell receptors to inhibit the immune function of cytotoxic T lymphocytes(CTLs) and regulate the change of IFN-γ (53). Tumor-derived lactate can down-regulate the level of nicotinamide adenine dinucleotide through translation, leading to autophagy defects, and then affect mitochondrial overactivation and reactive oxygen species(ROS) production, induce apoptosis of naive t cells, and reduce the antitumor immunity mediated by it (54).

Accordingly, the immunosuppressive properties of lactate may be limited to hypoxic conditions (55), where the hypoxic and acidified microenvironment created further complicates the effects of lactate on T-cell function. Lactate as a fuel can support the tricarboxylic acid(TCA) cycle regeneration of effector T cells, meet the energy supply of effector T cells, and promote the activation of effector T cells (56). Moreover, although the co-transport of lactic acid and H^+^ through MCT-1 and MCT-4 can prevent excessive intracellular acidification in activated T cells, the acidic environment tends to inactivate MCT isoforms and inhibit glycolysis rate through adverse feedback endpoints, ultimately inhibiting CD8^+^T cell activation (57). Newly presented evidence suggested that lactic acidosis can inhibit the production of IFN-γ by CD8^+^ T cells in CRC, and HIF-1α increases the transcription and enrichment of PTTG3P in the promoter region of CRC under the hypoxic microenvironment (58). MiR-1271-5p/PTTG3P/YAP1 axis promoted CRC cell proliferation and the production of glycolytic product lactate (**Figure 1**). The resulting aggravation of lactate acidification may further facilitate the proliferation and metastasis of CRC cells (58). It has also been confirmed that the accumulation of lactic acid produced by CRC cell metabolism and the acidified environment can not only inhibit the glycolysis and function of T cells (59) but also interferes with the translocation of NFAT to the nucleus (**Figure 1**). In this case, calcineurin regulates NFAT transport ASIC2-mediated acidosis through calcineurin/NFAT1 (C/N1) signaling (60, 61), reversal of the C/N1 pathway reduces the invasion and metastasis of CRC cells under acidosis by down-regulating ASCI2 expression (61).

### 2.2 NK cells

NK cells, first identified in 1975 by Herberman and Kiessling et al. (62, 63) are a distinct lymphocyte subset that arises primarily from standard lymphoid progenitor cells and contains characteristic cytoplasmic granules that constitute a third lymphoid cell in a MHC-I and antibody-independent manner. Tumors lacking MHC-I expression or carrying stress-induced proteins will cause the expression of NK cell-stimulating receptors in tumor cells to be stimulated by signals and then be expressed uncontrolled with ligands on tumor cells (64). Typically, another indirect way to kill tumors is through the immunomodulation of various immune cells (dendrites, macrophages, T cells, etc.) and their interaction to produce a variety of cytokines, growth factors, and chemokines (64). Cancer cells killed by NK cells can deliver tumor antigens to dendritic cells, triggering their maturation and differentiation (65). The production of lactic acid in CRC liver metastases reduces the pH value of the tumor microenvironment, and the excessively acidic tumor microenvironment can induce mitochondrial stress and apoptosis of liver-resident NK cells (66).

Mechanistically, lactate produced by tumor cells through glycolysis is exported to the extracellular space through MCT-1/4 and proton through the overexpression of GLUT1 and lactate transporter, and the *in vitro* acidic microenvironment induces endogenous ROS-mediated apoptosis of liver NK cells (67, 68). Thus, blocking mitochondrial ROS accumulation can effectively prevent NK cell apoptosis. Metabolic regulation measures aimed at restoring local NK cell activity and preventing tumor growth still have certain prospects and research value (66). Many lactic acids produced by tumor cell metabolism induce the expression of IFN-γ by inhibiting the up-regulation of NF-κb and NFAT signaling pathways (**Figure 1**), which inhibits NK cells and T cells, induces the apoptosis of immune cells, and leads to immune escape (69).

### 2.3 Tregs

Based on research findings, Foxp3^+^ Tregs are a subpopulation of lymphocytes with unique oxidative properties, which is essential for maintaining immune homeostasis and self-tolerance (70–72). They could regulate the function of various immune cells (lymphocytes, dendritic cells, macrophages, etc.) by secreting immunomodulatory cytokines and cytolytic molecules, and has a wide range of inhibitory activities (71, 73). Lactate may also activate CCL20/CCR6 axis by inducing the expression of TREM-1 in TAM cells, thereby attracting Treg aggregation and playing an immunosuppressive role (74, 75).

Lactate serves as a fuel to support gluconeogenesis and TCA cycle function in Treg cells (76). When effector and regulatory T cells are exposed to high lactate and low glucose, LDH reduces NAD^+^ to NADH. Still, the reduced NADH is not available for the NAD^+^ -dependent enzymatic reactions of GAPDH and PGDH. The targeted inhibition of glycolysis and serine production regulates T cell metabolism (77). Tregs suppress glucose metabolism by blocking the CD28 signaling pathway through CTLA-4 expression (78). The large amount of lactate produced by glucose deprivation in the TME also stimulates Foxp3 expression (**Figure 1**), allowing effector T-cells to differentiate into regulatory T cells. Foxp3 expression through Myc transcriptional repression helps Treg cells to regulate in a high L-lactate environment and simultaneously enhances NAD production and OXPHOS (79). Recently, Treg cells were indicated to promote the translocation of NFAT1 into the nucleus through MCT1 transport during lactic acidosis (**Figure 1**), and then upregulated the expression of PD-1 (80). More importantly, Alkbh5 might affect the efficacy of immunotherapy in CRC by regulating the expression of MCT4/Slc16a3 and the composition of lactate in TME, tumor-infiltrating Treg cells and MDSC cells, which has been identified as a potential immunotherapy target (81).

### 2.4 Monocytes, macrophages, and DCs

In sporadic CRC, M1 macrophages can accumulate in adenomas, but when malignant transformation into CRC occurs, M2 macrophages become the dominant macrophages (82). CRC cells mainly take advantage of the anti-inflammatory phenotype of M2 macrophages to inhibit tumor killing and immune surveillance (83). The pro-healing and matrix remodeling activities of M2 macrophages can promote tumor growth and metastasis (82).

DCs are often damaged in TME and play a critical role in initiating and promoting antitumor immune responses. Also, DC dysfunction can lead to immunosuppressive TME, and the highly immunosuppressive TME of CRC can affect the clearance and killing of tumors by the immune system (83). In response to lactic acidosis, monocytes differentiate into dendritic cells with an immunosuppressive phenotype (84, 85). They can promote CRC development by inducing the differentiation of TAMs in CRC to an M2-like phenotype through up-regulation of HIF-1α expression (86, 87). Lactate can also promote the differentiation of monocytes into macrophages with an inflammatory prototumer phenotype (31, 88). STAT6 phosphorylation in CRC may be an intrinsic key driving M2-type bone marrow-derived macrophages (BMDMs, Mφ)-M2φ polarization (89). The decrease of STAT6 phosphorylation can inhibit M2Φ polarization and down-regulate the expression of BRD4 and PD-L1 in M2Φ, thereby weakening the immunosuppressive effect of lactic acid in TME (90).

CircPCLE1 promotes glycolysis to produce lactate in CRC by regulating the miR485-5p/ACTG1 axis, which drives TAM M2 polarization (manifested as increased IL-10 and MRC1) and EMT (91, 92). MCT-1 absorbs lactate secreted by CRC cells and promotes gluconeogenesis and PGE2 production in THP-1 monocytes (93). Activated HIF-1α in THP-1 monocytes synthesized PGE2 and gluconeogenesis through the transcription of COX-2 and PEPCK, which ultimately promotes the growth of inflammation-related colorectal tumors (93). The interaction between tumor and TAM has long been considered related to TME, and lactate has recently been reported as an extracellular signaling molecule that regulates TAM polarization. Its elevated level is vital for the maintenance of TAMs activity (94, 95). Additionally, CRC progression has been reported to be associated with the expression of suppressor receptor signaling regulator alpha (Sirpα) in TAMs. CRC cell-derived lactate induces nuclear translocation of transcription factor Ap-2α from the cytoplasm of TAMs (**Figure 1**), which promotes Sirpα expression in TAMs by binding to the mouse Elk-1 promoter (96). Transgenic macrophages were specifically expressing Elk-1 regulate TAM phagocytic activity and CRC progression in a SIRPα-dependent manner (**Figure 1**), providing a potential target for macrophage immunotherapy in CRC patients (96). When excessive glucose consumption cannot give tumor cells continue to power the tumor microenvironment is in a state of severe hypoxia and lactic acid acidification, and TME elements such as CAFs and TAM and glutamine can replace glucose to CRC cell energy, transfer to maintain CRC cell proliferation activity (97).

## 3 Lactate homeostasis in CRC cells

CRC cells mainly use short-chain fatty acids such as butyric acid, propionic acid, and acetic acid as energy sources, which are converted to lactic acid through glycolysis to supply energy and energy conversion for CRC cells (98, 99). Lactic acid is a metabolite in the final stage of glycolysis. A variety of intestinal bacteria in the colorectal tumor microenvironment can produce lactic acid by fermentation (99). Regulation targeting these bacteria can also affect the proliferation and metastasis of CRC cells through metabolic reprogramming of lactic acidn (100). LDH can mediate the bidirectional conversion of pyruvate and lactate and is an important participant in the anabolism of lactate (101).

Interestingly, lactate efflux and uptake are dependent on the MCT transporter family. MCT-1 and sodium-coupled monocarboxylate transporter 1(SMCT1) are two major transmembrane transporters in enterocytes, located at the apical side of the intestinal epithelium (102). While cells are highly glycolytic, MCT is employed to transfer lactate and H^+^ from intracellular LDH, and MCH can regulate intracellular lactate and pH to prevent lactic acidosis (103). In the MCT family, MCT-4 can mediate lactate excretion by tumor cells or stromal cells, whereas MCT-1 mainly mediates lactate uptake by oxygen-containing cells in tumors (104). MCT-1 is not only found in cancer cells but also expressed in stromal cells such as fibroblasts and endothelial cells. As an important mediator of lactate uptake, MCT-1 can be upregulated on the plasma membrane of cancer cells of the lung, stomach, respiratory tract, colon, and nervous system (105). MCT-1 has long been identified as the major butyrate (BT) transporter in colonic epithelial cells. More importantly, lactate has been evaluated to play a dual role in CRC progression, with lactate export for cell survival and BT import for cell death (106). In addition to the synthesis and translocation of lactate, pathophysiological changes at other stages of glycolysis may also affect the last step of glycolysis, such as reprogramming metabolism of the metabolite lactate.

## 4 Effect of lactic acid on chemoresistance of CRC

**Figure 2:**
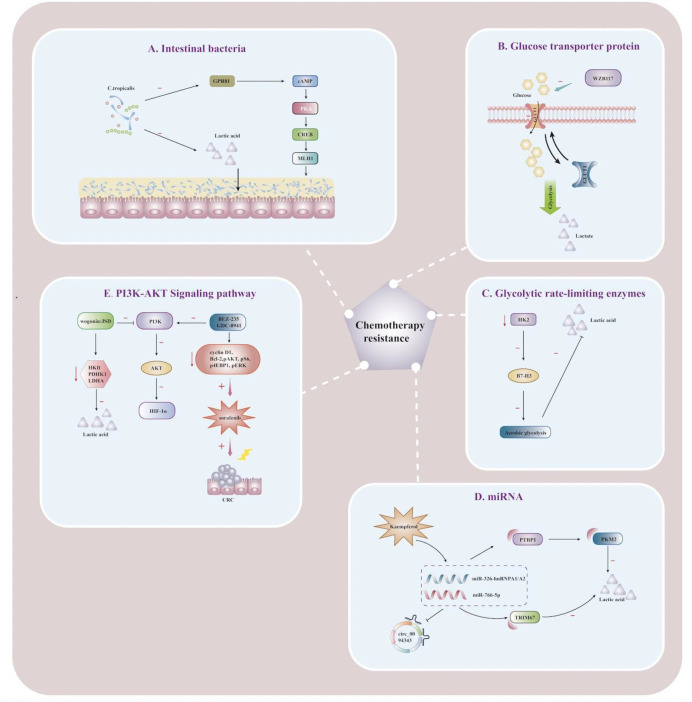
Lactate affects the immunometabolic mechanism of chemoresistance in CRC Schematic illustration showing mechanisms of action associated with targeting lactate metabolic reprogramming to reverse chemoresistance in CRC cells. **(A)** Schematic illustration showing the effect of lactic acid fermented by Clostridium tropicalis on chemotherapy tolerance in CRC patients. When Clostridium tropicalis produces large amounts of lactate, the GPR81-cAMP-PKA-CREB axis is activated, and MLH1 expression is downregulated, increasing chemotherapy tolerance. **(B)** Schematic illustration showing lactate regulation and reversal of chemotherapy resistance mediated by the mechanism of regulating gene expression by miRNA. circ_0094343 inhibits glycolysis of CRC cells through the miR-766-5p/TRIM67 axis, reduces lactate production, and enhances the sensitivity of chemotherapeutic drugs such as 5-FU. Dietary flavonoid kaempferol can inhibit lactate production and reverse chemotherapy resistance by regulating the miR-326-hnRNPA1/A2/PTBP1-PKM2 axis. **(C)** Schematic illustration showing the effects of glycolysis rate-limiting enzymes on lactate metabolism and drug resistance in CRC tumor microenvironment. When HK2 is inhibited, B7-H3-induced lactate and CRC chemotherapy resistance are reduced. **(D)** Schematic illustration showing the reversal of 5-FU resistance by PI3K/AKT pathway in CRC cells. Jiedu Sangen decoction (JSD) and wogonin can inhibit the PI3K/AKT pathway, down-regulate the expression of HIF-1α, reverse the chemoresistance, and increase the apoptosis of CRC cells. **(E)** Schematic illustration showing the effect of the glucose transporter GLUT1 on lactate metabolism and 5-FU resistance. WZB117 can specifically inhibit glucose transport by GLUT1, reduce glycolytic raw materials and lactate production, and improve 5-FU resistance. nosine monophosphate; PKA, protein kinase A; CREB, cAMP-response element binding protein; JSD, Jiedu Sangen decoction; HK2, hexokinase 2; MLH1, Recombinant MutL Homolog 1; PTBP1, Recombinant Polypyrimidine Tract Binding Protein 1;PKM2, Pyruvate kinase isozyme type M2; TRIM 67, Tripartite Motif Containing 67; HIF-1α, hypoxia-inducible factor-1α.

Microenvironmental hypoxia as well as abnormal HIF-1 activity in tumor cells are one of the causes of resistance to chemotherapy and/or radiotherapy in solid tumors (107–109).This is partly because most of the patients with CRC have problems of chemoresistance, recurrence and metastasis, it is necessary to explore the key targets and drugs to reverse resistance and improve the therapeutic efficacy. 5-FU and platinum are widely used in CRC, and recent studies have found that chemoresistance to 5-FU is the main reason affecting the therapeutic outcome of CRC patients (110). At the same time, the glycolytic phenotype is associated with tumor growth and chemoresistance, as mentioned above, targeting lactate metabolic reprogramming, whether signaling molecules or metabolites, maybe a potential pathway to reverse chemoresistance. The putative mechanisms of lactate in CRC chemotherapy resistance are summarized in **Figure 2**.

In general, patients with chemotherapy resistance are often accompanied by mismatch repair system (MMR) defects. Qu discoverd that lactic acid fermented by Clostridium tropicalis can enhance oxaliplatin tolerance in colon cancer patients, and targeted therapies that inhibit this metabolic pathway can significantly improve chemotherapy tolerance and MMR expression *in vivo *(111). Targeted inhibition of lactate production metabolism can significantly improve chemotherapy tolerance and reduce MMR expression. Furthermore, MLH1 expression could be down-regulated by the GPR81-cAMP-PKA-CREB axis to promote chemotherapy tolerance (**Figure 2**), and targeted inhibition of lactate production in C. tropicalis may improve the therapeutic effect (111). Increased levels of m^6^A and METTL3 were found in 5-FU resistant CRC cells, and targeted inhibition or knockdown of METTL3 could inhibit glycolysis in cancer cells and restore chemo-sensitivity to 5-FU in resistant CRC cells (112). The evidence highlighted that METTL3 enhanced LDHA expression, catalyzing the conversion of pyruvate to lactate, thereby promoting glycolysis and enhancing chemotherapy tolerance to 5-FU in tumor cells. METTL3/LDHA axis-induced glucose metabolism may be a potential therapeutic target for CRC cells to overcome 5-FU resistance (112), which was demonstrated to be associated with overexpression of GLUT1 in colon cancer cells. Inhibition of GLUT1 by the specific inhibitor WZB117 significantly increases the sensitivity of 5-FU-resistant cells to chemotherapeutic agents (**Figure 2**), providing an additional therapeutic option for patients with 5-FU-resistant colon cancer (113).

B7-H3, an immunoregulatory protein, is widely overexpressed in multiple tumor types and plays a vital role in tumor progression (114). Up-regulation of B7-H3 can promote glycolysis to produce large amounts of lactate by promoting the expression of HK2 in CRC cells, which is a crucial mediator of B7-H3-induced CRC resistance, and the expression of the two is positively correlated in tumor tissues. B7-H3 increased glucose consumption and lactate production by promoting HK2 expression in CRC cells, and HK2 was a key mediator of B7-H3-induced CRC chemoresistance (115). More importantly, targeting HK2 inhibitors can reverse B7-H3-induced increase in aerobic glycolysis and B7-H3-endowed chemoresistance of cancer cells. B7-H3 may become an emerging regulator targeting and inhibiting chemoresistance for CRC treatment (115). As a miRNA molecular sponge, circRNA can be involved in the pathophysiological processes of CRC cells, such as proliferation, migration, invasion, and apoptosis. Circ0094343 has been found to inhibit glycolysis and lactate metabolite production in CRC cells through the miR-766-5p/TRIM67 axis to play an antitumor role(**Figure 2**), while also enhancing the sensitivity to various chemotherapeutic drugs (5-FU, Oxaliplatin, and doxorubicin) to improve the efficacy of chemotherapy (116).

Dietary flavonoid kaempferol was found to overcome resistance to 5-Fu treatment by modulating the miR-326-hnRNPA1/A2/PTBP1-PKM2 axis to decrease lactate production during glycolysis in resistant CRC cells (**Figure 2**) (117). Jiedu Sangen decoction(JSD) may inhibit glycolysis and reverse 5-FU resistance through PI3K/AKT/HIF-1α signaling pathway (**Figure 2**), thereby inhibiting glycolysis, inducing apoptosis, and enhancing anti-tumor activity (118). Currently, wogonin has been regarded as a promising reversal agent for multi-drug resistance and may reverse cancer therapy resistance by inhibiting the PI3K/AKT pathway to downregulate HIF-1α expression and glycolytic flux (119). Also, wogonin could reverse drug resistance in HCT116 cells during hypoxia, which was mechanistically associated with down-regulation of the expression of glycolysis-related proteins (HKII, PDHK1, LDHA) and reduced lactate production (**Figure 2**) (120). The specific PI3K/AKT inhibitor LY294002 has been demonstrated by *in vitro* and *in vivo* experimental studies that the regulation of the PI3K pathway is associated with the tolerability and therapeutic effects of chemotherapeutic drugs such as 5-FU, paclitaxel, oxaliplatin, irinotecan, and adriamycin (121–125). Recently, LY294002 has been chiefly targeted with other medications, and the combination of chemotherapeutic drugs is used to overcome therapeutic resistance and enhance the killing impact on tumor cells. The combination of PI3K inhibitors BEZ-235 and GDC-0941 was found to have a synergistic effect on CRC cells under hypoxia. It could inhibit cyclin D1, Bcl-2, pAKT, pS6, p4EBP1, and pERK from reducing lactate release and increasing the antiproliferative effect of sorafenib (**Figure 2**) (126). The mTOR pathway highlights targeting tumor cell proliferation, survival, and metabolism to play a critical role in tumor development, treatment resistance, and poor prognosis (127). Excess lactate secreted by cancer cells undergoing metabolic reprogramming acts as a signaling molecule to modulate immune responses through extracellular acidification as an energy source by shuttling between different cell populations and is also a mechanistic target for inhibiting the mTOR pathway in immune cells (95). Therefore, how to overcome resistance to mTOR inhibitors in the future, as well as actively target the mTOR pathway using metabolic disturbances in cancer cells, may be of great help in the treatment of CRCs (128, 129).

## 5 CRC therapeutics targeting lactate production and translocation

### 5.1 Target lactate synthesis

As part of the tumor microenvironment, the gut microbiota has attracted much attention in recent years. A variety of intestinal commensals and their metabolites have been reported to trigger inflammatory cascades and oncogenic signaling, affecting the growth and spread of CRC (130), particularly these bacterial species, such as Peptostreptococcus anaerobic, Bifidobacterium, and Lactobacillus species (131–134). An intestinal mucosal barrier is a functional unit, and its inherent properties as a semipermeable barrier are essential for maintaining health. D-lactate, an oxygenated carboxylic acid produced by bacterial fermentation, is a valuable indicator of the integrity of the intestinal mucosal barrier (135). The gut mucosal barrier is a functional unit, and its inherent properties as a semi-permeable barrier are essential for maintaining health. D-lactate, an oxygenated carboxylic acid produced by bacterial fermentation, is a useful indicator for evaluating the integrity of the intestinal mucosal barrier (135). Lactococcus produced by lactic acid bacteria strains can synthesize antimicrobial peptides themselves, coupled with lactic acid bacteria fermentation to generate large amounts of lactic acid (**Table 1**). Acetic acid leads to acidification of the intestinal microenvironment, which together plays a role in inhibiting the growth of some pathogenic gram-negative bacteria in the intestine (136). Metagenics are substances that, unlike probiotics in the gut, are released by metabolic activities of microorganisms or are produced through microorganisms and can be applied as carcinogenic inhibitors (137). Lactate delivered by epigenetic supplements can maintain epithelial integrity by stimulating intestinal stem cell ISC proliferation through Wnt/ß-catenin signaling in Paneth cells and intestinal stromal cells, downregulating colonic inflammation-regulated immune tolerance by inhibiting YAP and NF-κB activation through GPR81, a cell surface receptor for lactate (138, 139).

**Table 1:** The most relevant pre-clinical and clinical trials of drugs targeting the regulation of lactate metabolism against CRCs.

Agents	Targets	Intervention mechanism	Functions	Stage	References
D-lactate	Oxygenated carboxylic acids produced by bacterial fermentation	Intestinal mucosal barrier dysfunction leads to bacterial translocation	Increased d-lactate levels are characteristic of intestinal mucosal barrier dysfunction in patients with early CRC	Preclinical study	135
Lactococcus	Lactic acid bacteria strains	Inhibition of invasion of Caco-2/TC7 cells by gram-negative bacteria	Antimicrobial peptides together with fermented lactic acid acetic acid lead to acidification of the intestinal microenvironment and inhibit the growth of some pathogenic gram-negative bacteria in the intestine	Preclinical study	136
Lactate	Epigenetic supplements	Wnt/ß-catenin signaling in Paneth cells and intestinal stromal cells, and GPR81-related signaling pathways inhibit YAP and NF-κB activation	Maintaining epithelial integrity by stimulating intestinal stem cell ISC proliferation through Wnt/ß-catenin signaling in Paneth cells and intestinal stromal cells, and downregulating colonic inflammation-regulated immune tolerance by inhibiting YAP and NF-κB activation through GPR81-related signaling pathways	Preclinical study	138, 139
Epigallocatechin gallate	——	Circular Ribonucleic Acid Actin Gamma 2/Micro Ribonucleic Acid-370-5p Pathway	Decreasing CRC cell viability by inhibiting angiogenesis and glycolytic lactate production in CRC	Preclinical study	144
Atractylenolide	——	AKT/mTOR signaling pathway	Decreasing lactate production during the Warburg effect	Preclinical study	148
IDF-11774	HIF-1 inhibitor	Targeting HSP70, inhibiting mitochondrial respiration, activating AMPK, and downregulating HIF-1α expression	Inhibiting extracellular acidification rate (ECAR) and oxygen consumption rate (OCR)	Preclinical study	146, 147
LY294002	PI3K/AKT inhibitor	PI3K / AKT / mTOR signaling pathway	Impacting CRC cell growth and glucose metabolism by silencing KLK10	Preclinical study	151
Rapamycin	mTOR inhibitor	PI3K / AKT / mTOR signaling pathway	Reducing lactate production by aggravating KLK10 gene knockdown and inhibiting mTOR-mediated HIF-1α activation	Preclinical study	152
P. freudenreichii	Enteric microorganism	Stimulated by extracellular lactate in CRC to promote the "Warburg effect"	The increase of short-chain fatty acids such as acetic acid and propionic acid can not only enhance the cytotoxicity to CRC cells but also play a certain immunosuppressive role and reduce the risk of inflammatory CRC by promoting histone acetylation and regulating the transcriptional activity and immunoregulatory genes of various tumor suppressors	Preclinical study	105, 151, 152
Fusobacterium nucleatum	Enteric microorganism	Decreasing levels of Toll-like receptor 4 signaling to MYD88, leading to activation of the nuclear factor-κB, increased expression of miR21, and RAS GTPase RASA1 by activating Toll-like receptor 4 signaling to MYD88, leading to activation of the nuclear factor-κB	Patients with higher tissue Fusobacterium nucleatum DNA and miR21 levels are, therefore, more likely to have a poor prognostic survival outcome	Preclinical study	152
AZD3965	A selective inhibitor of MCT-1	Increasing TCA cycle-related metabolites,13c-glucose mitochondrial metabolism enhanced oxidative pyruvate dehydrogenase and anaplastic pyruvate carboxylase flux	The increase of the bioenergy of HT29 cells by causing an increase in circulating metabolic intermediates in the TCA cycle in MCT-4 ^+^ HT29 cells	Phase 1 clinical trials	156, 157
Dickkopf2(DKK2)	——	VEGF/VEGFR independent pathway, activating downstream mTOR signaling pathway: via demethylation of miR-493-5p in an autocrine or paracrine manner	Accelerating aerobic glycolysis in CRC cells and secretes more lactate to stimulate angiogenesis. Stimulating CRC progression	Preclinical study	159
7ACC2	Inhibitor of MCT	Increasing PH in the microenvironment by blocking lactate trafficking from tumor cells, inhibiting Human Umbilical Vein Endothelial Cells tube formation	Increasing PH in the microenvironment by blocking lactate trafficking from tumor cells, inhibit Human Umbilical Vein Endothelial Cells tube formation	Preclinical study	159
Lonidamine	Inhibitors of MCT-1, MCT-2, and MCT-4	Enhancing glutamine catabolism	Inhibiting lactate transport, leading to intracellular lactic acidosis and promoting other metabolic pathways (e.g., glutamine catabolism) to compensate for reduced glycolytic flux	Preclinical study	160, 161
α-cyano-4-hydroxycinnamic acid, 4,4'-diisothiocyanatostilbene-2,2'-disulphonic acid, and quercetin	Inhibitor of MCT	Disrupting the glycolytic phenotype	Increasing intracellular lactate content and induced tumor cell death in CRC cells	Preclinical study	162
WZB117	GLUT1 inhibitors	Stimulating platelet-derived growth factor	Increasing glycolysis, intracellular lactate content, and other acidic metabolites	Preclinical study	165, 166
Oridonin	Active diterpenoid	Rapidly inactivate p-AMPK, down-regulate the expression of GLUT1 and MCT-1	Inhibiting glucose uptake, reducing lactate output, and inducing autophagy and death in CRC cells	Preclinical study	167, 168
Dioscin	——	Enhancing the binding of the E3 ligase FBW7 to c-myc, which helps to inhibit HK2	Inhibiting glycolysis in CRC cells to exert antitumor activity	Preclinical study	172
Benz	A selective HK2 inhibitor	Inhibiting HK2 enzymatic activity	Decreasing lactate production and leading to increased apoptosis and mitochondrial membrane potential loss	Preclinical study	173
Epigallocatechin-3-gallate	Phosphofructokinase inhibitor	Up-regulating P53 gene and regulating NF-κB, JAK/STAT3 pathway, and inhibiting PFK activity	Reducing the rate of lactate production by glycolysis	Preclinical study	175–178

**Figure 3:**
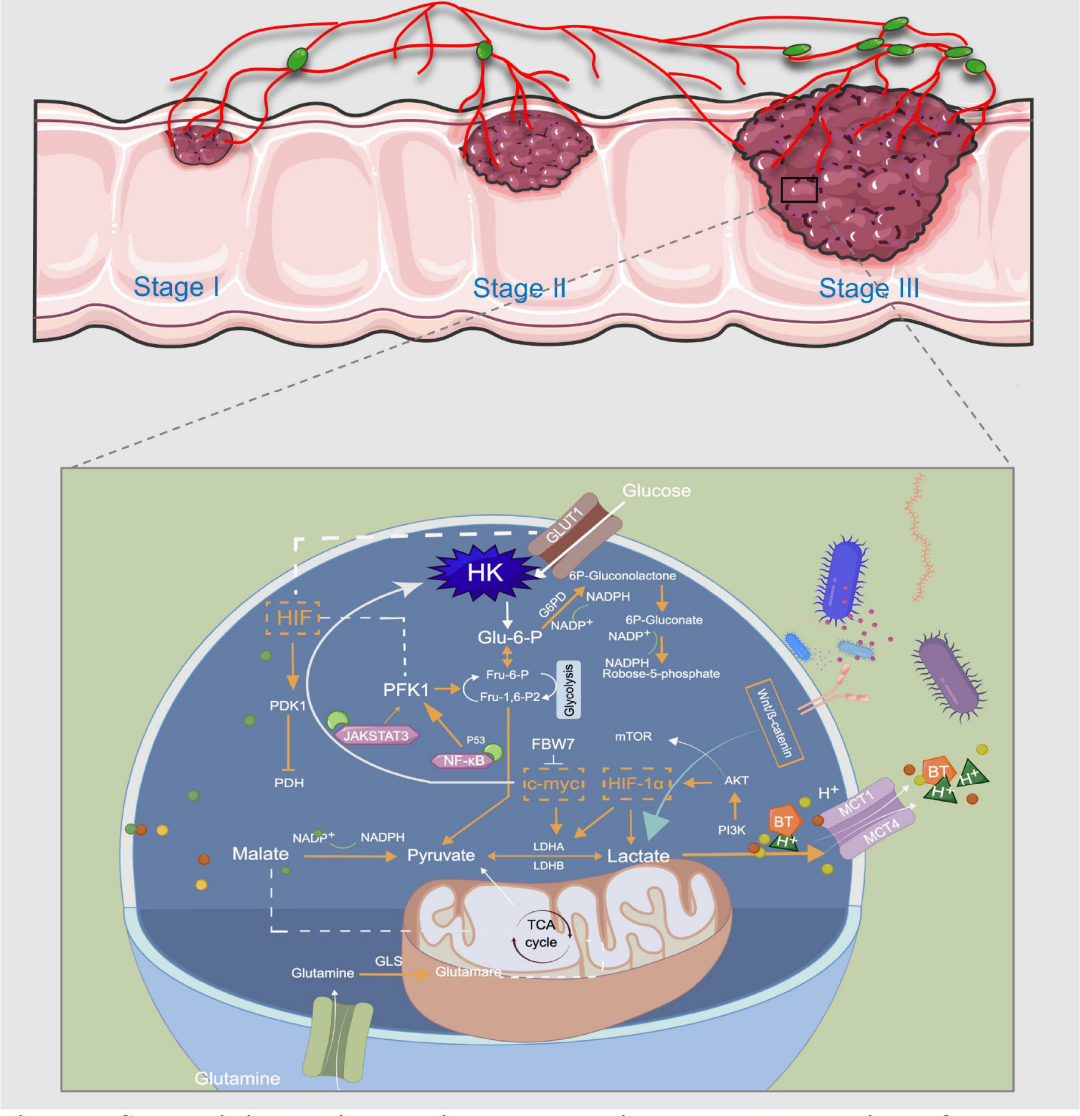
Schematic illustration showing the metabolic regulatory mechanisms of lactate involved in CRC treatment. Usually, the last step in glucose metabolism in cancer cells is aerobic glycolysis, which efficiently breaks down the produced lactic acid. GLUT1 transports glucose from the extracellular microenvironment into the cell and generates pyruvate through a series of reactions. This is the first stage of glycolysis, and a variety of enzymes, such as HK and PFK, are the rate-limiting enzymes in this stage. LDHA and LDHB can mediate the bidirectional conversion of pyruvate and lactate, and sustained activation of HIF-1a, c-Myc, and mTOR pathways induce abnormal expression of multiple glycolytic enzymes, thereby promoting aerobic glycolysis of cancer cells. MCT-1 can mediate BT transport, and acetate promotes lactate production through the plasma membrane relocalization of MCT-1. Glutamine and pyruvate participate in the TCA cycle in mitochondria for oxidative energy supply. Lactate controls CRC metabolic reprogramming in multiple steps to meet the metabolic energy demands of cancer cell proliferation and metastasis. This image was drawn by Figdraw.

In recent years, LDH has emerged as an emerging anticancer target and can mediate the bidirectional conversion of pyruvate and lactate. It is a tetramer composed of two different subunits, LDHA and LDHB (101). LDHA is the predominant isoform found in skeletal muscle and other highly glycolytic tissues that catabolizes pyruvate to lactate and produces NAD^+^ (**Figure 3**). In contrast, LDHB converts lactate to pyruvate or uses lactate as a nutrient source for oxidative metabolism or gluconeogenesis (140). Transcription factors HIF-1α and c-Myc can up-regulate the expression of LDHA gene and inhibit the expression of LDHB gene from maintaining higher glycolytic activity and produce more lactate (141). LDH inhibitors have been demonstrated to significantly increase radiosensitivity associated with G2/M cell cycle arrest by downregulating the expression of heat shock proteins such as HSP70 by increasing extracellular lactate/pyruvate concentrations, and pharmacological targeting of radiotherapy combined with LDH in the future can reduce radiotoxicity to normal tissues while increasing tumor-killing ability (142). N-Hydroxyindole-based inhibitors are small molecule inhibitors that compete with pyruvate and NADH and exhibit good antiproliferative and starvation-inducing abilities in various cancers (143). Interestingly, the clinical use of LDH inhibitors is limited by the interaction of other drugs with low selectivity or complex cellular components, and the heterogeneity of LDH expression between different cancers requires more cell type-specific studies in this field. Epigallocatechin gallate inhibited CRC angiogenesis and lactate production in glycolysis through Circular Ribonucleic Acid Actin Gamma 2/Micro Ribonucleic Acid-370-5p Pathway and reduced CRC cell viability (**Table 1**) (144).

Metabolic reprogramming, widespread in cancer cells, is balanced oxygen supply by HIF-1 as a master regulator and coordinated at the transcriptional level. HIF-1 also increases oncogene activity through the inactivation of tumor suppressors (e.g., VHL) and through PI3K/AKT/mTOR in cancer cells, ultimately driving cancer progression and metabolic alterations that are resistant to treatment (145). IDF-11774 is a HIF-1 inhibitor that targets HSP70 and inhibits mitochondrial respiration, activates AMPK and downregulates HIF-1α expression in HCT116 cells under hypoxia, and inhibits extracellular acidification rate (ECAR) and oxygen consumption rate (OCR) in cancer cells by downregulating HIF-1α target gene expression reducing glucose uptake, lactate levels, and NAD^+^ and NADP^+^ contents (**Table 1**) (146, 147). Although there are many *in vitro* and *in vivo* studies of HIF-1 inhibitors, only a few have reached clinical trials, and no clinical trials for CRC can be used as a clinical reference. Atractylenolide was validated to inhibit CRC cells proliferation and invasion by generating ROS through the AKT/mTOR signaling pathway and decreasing lactate production during the Warburg effect (148). Consistently, PI3K/AKT inhibitor LY294002 or mTOR inhibitor Rapamycin will inhibit CRC progression by aggravating KLK10 gene knockdown and inhibiting mTOR-mediated HIF-1α activation and decreasing lactate production (**Table 1**). Such results suggest that KLK10 silencing can affect CRC cells growth and glucose metabolism through PI3K/AKT/mTOR signaling and may become a potential target to support CRC treatment in the future (149, 150).

### 5.2 Target lactate transport

P. freudenreichii uses lactate in colon cells as an ideal carbon source for producing hyperpropionic acid and acetic acid, which oxidizes to pyruvate and then reduces to propionic acid (**Table 1**) (151). Typically, the increase of short-chain fatty acids such as acetic acid and propionic acid can not only enhance the cytotoxicity to CRC cells but also play a particular role in immunity by promoting histone acetylation and regulating the transcriptional activity and immunoregulatory genes of various tumor suppressors, thereby reducing the risk of inflammatory CRC (105, 152). It has been confirmed that infection of CRC cells with Fusobacterium nucleatum can increase their invasion and proliferation activity and xenograft tumorigenicity in mice (152). Mechanistically, Fusobacterium nucleatum decreased Toll-like receptor 4 signaling to MYD88, leading to activation of the nuclear factor-κB, increased expression of miR21, and RAS GTPase RASA1 by activating Toll-like receptor 4 signaling to MYD88, leading to activation of the nuclear factor-κB. Patients with higher tissue Fusobacterium nucleatum DNA and miR21 levels are, therefore, more likely to have a poor prognostic survival outcome (152).

Hence, Fusobacterium nucleatum can be used as a probiotic to regulate the conversion of lactic acid and short-chain fatty acids to regulate immune responses in the prevention or treatment of colon cancer. MCT-1 mediated transport of 14C-BT in Caco-2 cells is regulated by acute or chronic exposure to some drugs (indomethacin, acetaldehyde, caffeine, theophylline) and drugs of abuse (tetrahydrocannabinol and 3, 4-methylene dioxymethamphetamine) (153–155). Acetaldehyde, structural analogs of butyric acid such as indomethacin, competitively inhibit the uptake of butyric acids, such as acetate promoting plasma membrane relocation of MCT-1 and further triggering metabolic changes such as increasing glucose consumption and lactate production, thereby increasing the glycolytic phenotype of CRC cells (105). AZD3965, as a selective inhibitor of MCT-1 in CRC therapy, may cause an increase in circulating metabolic intermediates in the TCA cycle in MCT-4^+^ HT29 cells, which is further converted into bioenergy in HT29 cells(**Table 1**) (156, 157). In addition, AZD3965-induced substitution of SR13800, an inhibitor of MCT-1 in Raji cells, reported a decrease in bioenergetics and levels of tricarboxylic acid cycle intermediates accompanied by lactate accumulation (156, 157). These findings exploit the potential to combine MCT-1 inhibitors with mitochondrial-targeted therapies clinically. AZD3965 is currently in Phase 1 clinical trials and is entering the expansion phase of the trial. Understanding how it acts on cellular metabolism and identifying pharmacodynamic(PD) biomarkers for MCT-1 regulation are necessary to develop further and promote this combination of drugs and other mitochondrial-targeted therapies (156).

DKK2 is a secreted protein highly expressed in metastatic CRC tissues. It can stimulate angiogenesis by accelerating aerobic glycolysis and secreting more lactate in CRC cells through the classical VEGF/VEGFR independent pathway or activating the downstream mTOR signaling pathway (158). In addition, DKK2 stimulates CRC progression by initiating the demethylation of miR-493-5p in an autocrine or paracrine manner through a novel VEGF-independent, but energy-metabolism-related pathway (159). In these cases, DKK2 may be a potential anti-angiogenic target in treating patients with advanced CRC (159). 7ACC2, as an inhibitor of MCT, can increase PH in the microenvironment by blocking lactate trafficking from tumor cells, inhibit Human Umbilical Vein Endothelial Cells tube formation, reduce subcutaneous tumor growth, and ultimately inhibit CRC progression and metastasis *in vivo* and vitro (159).

LND acts as a common inhibitor of MCT-1, MCT-2, and MCT-4, inhibiting lactate transport, leading to intracellular lactic acidosis, and promoting other metabolic pathways (e.g., glutamine catabolism) to compensate for reduced glycolytic flux (160, 161). Amori et al. have found that α-cyano-4-hydroxycinnamic acid, 4,4’-diisothiocyanatostilbene-2,2’-disulphonic acid, and quercetin disrupt the glycolytic phenotype and induce tumor cell death by targeting the inhibition of MCT (162). In addition, these MCT inhibitors combined with 5-FU application can enhance the cytotoxicity of 5-FU and enhance the anticancer effect because they inhibit the proliferation of CRC cells (162). MCTs responsible for extruding lactate into the extracellular space may play a key role in CRC development. The application of these transporters combined with conventional treatment of CRC is expected to be a potential new therapeutic target. Therefore, further investigation is warranted to understand how LND drugs can be combined with chemotherapy and physical therapy in the future to improve selectivity for lesion tissues and maintain low toxicity in normal tissues (163).

### 5.3 Target glucose transport

GLUT1, as a human glucose transporter, can transfer glucose into cells and metabolize it to pyruvate through glycolysis for further reduction to form lactate (164). GLUT1, as a target gene of HIF-1, is involved in the regulation of glucose metabolism together with LDHA and MCT-4, and some studies have found that even in the presence of GLUT1 inhibitors (such as WZB117), if platelet-derived growth factor is stimulated, it increases glycolysis, intracellular lactate content and other acidic metabolites in colon cancer cells and promotes tumor progression (**Table 1**) (165, 166). Oridonin is an active diterpenoid isolated from Rabdosia rubescens in the 1970s (167), which can rapidly inactivate p-AMPK, down-regulate the expression of GLUT1 and MCT-1 while inhibiting glucose uptake, reduce lactate output, and induce autophagy and death in CRC cells. Therefore, it can be used as a glucose metabolism targeting agent for CRC therapy (168). Oridonin has been primarily investigated individually and in combination over the past decade. Future studies of resistance during or after reversal therapy can be targeted, as well as continuing to explore its novel biological mechanisms and functions in inflammation and immune regulation (169).

### 5.4 Targeting rate-limiting enzymes in glycolysis

The three rate-limiting enzymes in glycolysis are HK, PFK1 and PK, and their reactions are irreversible (**Figure 3**). HK is the rate-limiting enzyme in the first stage of glycolysis, and its expression level helps to distinguish cancer cells from normal cells, which is a novel antitumor therapeutic target (170, 171). HK2 is most closely related to malignant tumors, and dioscin has been shown to promote ubiquitination and degradation of c-myc by enhancing the binding of the E3 ligase FBW7 to c-myc, which helps to inhibit HK2 and inhibit glycolysis in CRC cells to exert antitumor activity (172). Benserazide (Benz), a selective HK2 inhibitor, significantly inhibited HK2 enzymatic activity *in vitro*, decreased lactate production and intracellular ATP levels, and may lead to increased apoptosis and mitochondrial membrane potential loss (173). Li further conducted *in vivo* studies on the preparation of benserazide nanoparticles. The results showed that 100 and 200mg/Kg of lipofectamine benserazide had potent inhibitory results on SW480 cell xenograft mice. Intraperitoneal injection of Benz at 300 and 600mg/Kg inhibited cancer growth in tumor-bearing mice without showing toxic effects (173). From a composite library of 8871 clinically used and well-annotated pharmacological compounds, Druzhyna et al. identified benserazide as an antitumor agent that inhibits colon cancer cell proliferation by reducing Cystathionine-β-synthase (CBS) activity in HCT116 and HT29 cell lines with high CBS expression. Such results suggest that benserazide might be a potential candidate for the experimental treatment of CRC. However, further pharmacokinetic and pharmacodynamic studies, preclinical studies, and clinical trials are warranted to evaluate the therapeutic potential of benserazide in CRC (174). Phosphofructokinase (PFK) acts on the rate-limiting enzyme of fructose-6-phosphate, and regulating of this enzyme is also an essential step in regulating glycolysis. Epigallocatechin-3-gallate is a safe and influential component in green tea and can induce CRC cell apoptosis by up-regulating the P53 gene and regulating NF-κB, JAK/STAT3 pathway, and inhibiting PFK activity, reducing the rate of lactate production by glycolysis (175–178). The most pre-clinical and clinical trials of drugs targeting the regulation of lactate metabolism against CRCs are showed in **Table 1**.

## 6 Conclusion and prospect

It was well known that cancer cells can reprogram their own and neighboring stromal cell metabolic pathways, and the more common glycolytic processes are often deregulated to meet accelerated bioenergetic and metabolic demands. Cancer therapy based on the Warburg effect requires targeting cancer-specific glycolytic targeting. As highlighted in this review, lactate plays an important role in colorectal carcinogenesis and accelerated progression, while lactate research has been at the forefront of defining mechanisms that integrate nutritional signaling into metabolite fluctuations, immune cell plasticity, and apparent modification of cancer. Intestinal microflora in the human intestinal microenvironment as, a component of TME, can ferment and generate lactic acid to regulate CRC cell activity. LDH is one of the main enzymes connecting tumors and stroma, which can affect the interaction between CRC and stroma through the generation of lactic acid. The monocarboxylate transporter family is a crucial transmembrane carrier, of which only MCT-1, MCT-2, and MCT-4 are involved in lactate transfer in tumor cells. They try to block the final stage of glycolytic synthesis in cancer cells by targeting lactate synthesis and transport, which can be used as a new therapeutic target for CRC. But MCT inhibitors also have shortcomings such as 1) lack of persuasive pharmacodynamic trials and drug toxicity studies, 2) weak selection specificity, and 3) combined with other targets, clinical trials related to efficacy evaluation of chemoradiotherapy, which requires further more meticulous studies. Targeting other stages of glycolysis, such as the level of regulatory rate-limiting enzyme activity, can also affect the energy supply of cancer cells, and lactate flux in the TME may help design future complementary therapies. More importantly, targeted lactate metabolism reprogramming has the potential to be a potential pathway to reverse chemoresistance and increase treatment sensitivity, and METTL3/LDHA axis-induced glucose metabolism may be a potential therapeutic target for CRC cells to overcome 5-FU resistance (112). WZB117, a specific inhibitor of GLUT1, could shed light on a drug-resistant colon cancer patient because it could promote the chemotherapeutic effect of 5-FU. The MCT1 inhibitor AZD3965 is considered to have the potential to combine mitochondrial targeting therapy, most likely because it has been shown to block pyruvate-lactate transport and activate mitochondrial metabolism in the presence of large increases in lactate (156). Moreover, AZD3965 is currently in phase I clinical trials and is progressing to phase II clinical trials. In order to further evaluate the therapeutic effect and safety of drugs targeting lactate metabolism reprogramming in CRC, more *in vivo* evidence needs to be presented, and multi-center clinical trials with expanded scope are necessary.

Currently, the epigenetic regulation of lactate on CRC genes is still in its infancy, so it will be interesting to comprehensively define how lactate metabolism in CRC affects epigenetic programming under different TME conditions. Lactate-related metabolites, immune cells, and matrix environment regulation form a close relationship network. The upcoming results may provide clinical solid evidence to explore effective mode of lactate-related metabolic enzyme inhibitors in combination with other drugs and the mechanism of treatment resistance, which may contribute to treatment strategies and survival benefits in CRC.

However, adverse reactions, dose-limiting toxicity, low stability, limited bioavailability, and non-specific pharmacological action distribution in the treatment of CRC create several challenges (179). For instance, nanotechnology has been tried to be applied in the treatment of CRC because of its good biocompatibility, biodegradability, and unique carrier advantages of targeted delivery as a hotspot of pharmaceutical technology (180). Polylactide-glycolic acid (PLGA) is an aliphatic polyester with a wide range of biomedical applications. In terms of material composition, PLGA is a random copolymer composed of lactic acid and glycolic acid, which has been approved by the FDA as an effective carrier for drug delivery and a scaffold for tissue engineering (181). Current evidence suggests PLGA nanoparticles as specific delivery vectors to load chemotherapeutic drugs such as 5-FU and oxaliplatin, which improve the proliferation inhibition and apoptosis induction effect on CRC cells and improve the efficacy of chemotherapy (182–186). Combining PLGA nanotechnology polymerized with other modern medical strategies can play a role in synergizing and reducing treatment tolerance and contribute to rational new drug development for CRC. Unfortunately, PLGA has not yet been popularized due to its high cost and professional preparation technology, which remains to be further improved.

## Author contributions

JL conceived the review. QS, JW, and GZ critically analyzed the current literature and wrote the original manuscript. TL, XZ, BN, BX, and XM contributed to the figures and revised the review. All authors have read and approved the final manuscript.
